# Great Genetic Differentiation among Populations of *Meconopsis integrifolia* and Its Implication for Plant Speciation in the Qinghai-Tibetan Plateau

**DOI:** 10.1371/journal.pone.0037196

**Published:** 2012-05-10

**Authors:** Fu-Sheng Yang, Ai-Li Qin, Yu-Fei Li, Xiao-Quan Wang

**Affiliations:** State Key Laboratory of Systematic and Evolutionary Botany, Institute of Botany, the Chinese Academy of Sciences, Beijing, China; University of Oxford, United Kingdom

## Abstract

The complex tectonic events and climatic oscillations in the Qinghai-Tibetan Plateau (QTP), the largest and highest plateau in the world, are thought to have had great effects on the evolutionary history of the native plants. Of great interest is to investigate plant population genetic divergence in the QTP and its correlation with the geologic and climatic changes. We conducted a range-wide phylogeographical analysis of *M. integrifolia* based on the chloroplast DNA (cpDNA) *trn*L-*trn*F and *trn*fM-*trn*S regions, and defined 26 haplotypes that were phylogenetically divided into six clades dated to the late Tertiary. The six clades correspond, respectively, to highly differentiated population groups that do not overlap in geographic distribution, implying that the mountain ranges acting as corridors or barriers greatly affected the evolutionary history of the QTP plants. The older clade of *M. integrifolia* only occurs in the southwest of the species' range, whereas the distributions of younger clades extend northeastward in the eastern QTP, suggesting that climatic divergence resulting from the uplift of the QTP triggered the initial divergence of *M. integrifolia* native to the plateau. Also, the nrDNA ITS region was used to clarify the unexpected phylogenetic relationships of cpDNA haplotypes between *M. integrifolia* and *M. betonicifolia*. The topological incongruence between the two phylogenies suggests an ancestral hybridization between the two species. Our study indicates that geographic isolation and hybridization are two important mechanisms responsible for the population differentiation and speciation of *Meconopsis*, a species-rich genus with complex polyploids.

## Introduction

The evolutionary history of plant species could be dramatically influenced by habitat fragmentation [1], [2]. The process of fragmentation would strongly reduce the dispersal ability of individuals among resource patches, leading to the breakup of a large, genetically variable population into isolated small subpopulations [3]. An increasing number of studies indicated that the rapid decline of population size and restricted gene flow could result in genetic differentiation between populations and genetic diversity loss, giving rise to species extinction and biodiversity decrease [1], [2], [4]. On the other hand, both evolutionary theory and empirical data indicate that global genetic variation could be maintained or even increased by a fragmented population, and allopatric speciation is prone to occur due to genetic isolation of fragmented populations [5], [6]. However, the genetic and ecological consequences of habitat fragmentation depend critically on the levels of gene flow between habitat fragments [2], [3], [4]. So the analysis of genetic divergence and gene flow between fragmented populations would be greatly helpful in understanding the process of speciation or extinction under the effects of environmental changes.

**Figure 1:**
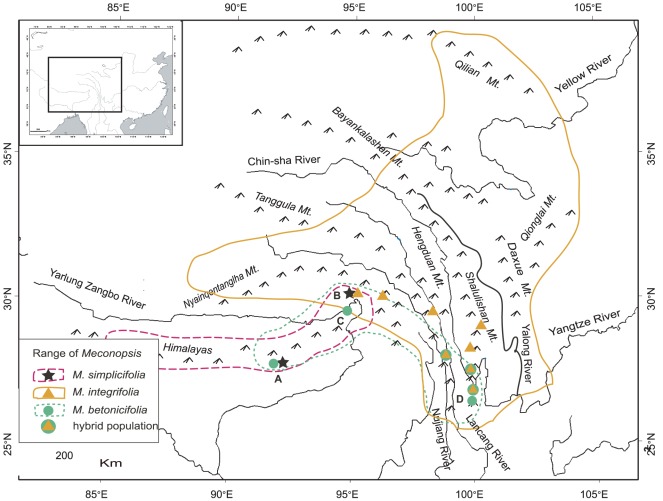
A map showing the species' ranges and a scenario of possible hybridization between species. Letters A–D indicate the sampling sites for *M. simplicifolia* (star) and *M. betonicifolia* (filled circle), A: Cuona, B: Linzhi, C: Milin, and D: Laojunshan Mountain. Triangle, solid circle, and pentagram represent some sampled populations of *M. integrifolia*, *M. betonicifolia*, and *M. simplicifolia*, respectively. Putative hybrid populations are indicated by the overlapping of solid circle and triangle.

The phased uplift of the Qinghai-Tibetan Plateau (QTP) continuing from the middle Tertiary has greatly changed the geology and topography of East Asia, leading to the unique geomorphological configuration and complex land conditions [7]. The highest plateau and mountains ranges have been incised deeply by numerous valleys or rivers (Fig. 1), resulting in the rapid development of endemic, specialized montane species along mountain ranges and the concentration of the relatively old Tertiary flora in isolated low valleys [8], [9]. It would be of particular interest to unravel the roles of geographic and ecological changes in the species diversification by tracing the genetic footprint of environmental shifts in the QTP plants. Phylogeography, the analysis of the spatio-temporal pattern of population genetic variation, has been proved to be very efficient in retrieving the evolutionary history of species or close relatives on a relatively short evolutionary timescale [10], [11].

To date, the geographic patterns of population genetic variation were investigated on more than ten plant species from different lineages in the QTP [12]. The results clearly indicated the effect of the Quaternary climatic oscillations on the development of geographic genetic structure [12], [13], [14], and the recolonization patterns from multiple refugia on the QTP were also outlined. At the same time, the discovery of some putative refugia or micro-refugia on the plateau implied that some of the populations might have survived the Last Glacial Maximum or even earlier glaciations in situ [13], [14]. Also, the time of lineage divergence within some species could be dated to the late Tertiary [15], [16], [17], when the dramatic uplift of the QTP occurred [7]. However, compared with the well-discussed population evolutionary dynamics driven by the Quaternary climatic oscillations [12], much less attention was paid to the effects of tectonic events on plant divergence and speciation in the QTP [6], [14], [18].

The genus *Meconopsis* comprises about 50 species that are mainly distributed in the QTP and the neighboring mountains, with the only European species *M. cambrica* inhabiting in the humid and shady deciduous forest from Ireland, south-west England to Northern Spain [19]. *Meconopsis integrifolia*, one of the most widely distributed species of the genus, is a flagship species of the alpine scree in the QTP. It is scattered in the scree along mountain ridges, with an island-like distribution at altitudes of 4000–5200 m above sea level. In the eastern edge of its range, the species often grows in patchy habitat under azaleas at lower altitudes of 2700–4000 m above sea level. The bright yellow flower of this species makes it easy to be distinguished from the blue (or sky blue) flower relatives *M. betonicifolia* and *M. simplicifolia*, two quite narrowly distributed species in the southeast QTP (Fig. 1). A range-wide phylogeographical analysis of *M. integriflolia* and its close relatives is very helpful to reveal the underlying mechanism of the habitat fragmentation and to explore the genetic and ecological consequences of environmental changes for the QTP plants.

In the present study, the cpDNA *trn*L-*trn*F and *trn*fM-*trn*S regions were used to detect genetic variation and gene flow through seed between the highly fragmented populations of *M. integrifolia* and to retrieve the phylogeographical history of the species. Since the phylogenetic analysis on the two cpDNA markers indicated that one clade of *M. integrifolia* was nested within *M. betonicifolia*, it is interesting to know whether hybridization or ancestral polymorphism is responsible for it. Different from the uniparentally inherited cpDNA markers, the nuclear genes are biparentally inherited, with a rich source of phylogenetic information, so we tried to isolate some single/low copy nuclear genes to investigate phylogenetic relationships between the species and its relatives. However, complex polyploidization is quite prevalent in *Meconopsis* [20] and it is difficult to determine orthology/paralogy of the genes. The internal transcribed spacer (ITS) of nuclear ribosomal DNA (nrDNA) is tandemly repeated with hundreds to thousands of copies, which could be homogenized by concerted evolution that eliminates sequence variation among the different copies [21]. Therefore, the nrDNA ITS region was also used to retrieve the phylogenetic relationship of *M. integrifolia* and its close relatives.

## Materials and Methods

### Ethics statement

No special permits were required for this study because all samplings were collected by researchers with introduction letters of IBCAS (Institute of Botany, Chinese Academy of Sciences) in Beijing.

### Population sampling

Population sampling was conducted throughout the range of *Meconopsis integrifolia* during the summers of 2005–2009. Fresh leaves were collected from 35 populations and, with few exceptions, 15–29 individuals were sampled from each population (Table S1). To avoid sampling closely related individuals, the samples were at least 30 m apart from each other. In total, 757 individuals of *M. integrifolia* were sampled and leaves were dried with silica gel. In addition, fifteen individuals of *M. betonicifolia* were sampled from Cuona and Milin Counties of Tibet, and the Laojunshan Mountain in Yunnan Province. These three populations can represent the easternmost, middle and westernmost distribution of the species, respectively (Fig. 1). Ten individuals of *M. simplicifolia* were sampled from Linzhi and Cuona Counties in Tibet, representing its easternmost and Himalayan populations, respectively. Also, four individuals from two populations of *M. chelidonifolia* were sampled as outgroups. The latitude, longitude and altitude of each collection location were measured using an eTrex Global Positioning System (Garmin).

### DNA extraction, PCR amplification, cloning and sequencing

Total genomic DNA was extracted from silica gel-dried leaves using the modified CTAB method [22] and used as template in the polymerase chain reaction (PCR). Two cpDNA intergenic spacers, *trn*fM-*trn*S [23] and *trn*L-*trn*F [24], were amplified and directly sequenced for all samples. Amplification was conducted in a Tpersonal thermocycler or Biometra T1 themocycler (Biometra, Goettingen, Germany). The PCR products were purified using Gel Band Purification Kit (Tiangen), and then sequenced using the ABI Prism Bigdye Terminator Cycle Sequencing Ready Reaction Kit on an ABI PRISM 3730xl analyzer. To investigate the evolutionary history of the *M. integrifolia* clade that was nested within *M. betonicifolia* in the cpDNA phylogeny, 26 individuals of *M. simplicifolia*, *M. betonicifolia* and *M. cheilanthifolia* and 151 samples from 20 populations that can represent the seven geographic groups (Table S1) of *M. integrifloia* were further used in the nrDNA ITS analysis. The nrDNA ITS region was amplified and sequenced with the primers ITS1 and ITS4 of White *et al*. [25]. However, one or more double-peaks were detected in the chromatograms of 17 individuals. All these individuals, and four additional ones without double-peaks in the chromatograms, were cloned with the pGEM-T Easy Vector System II (Promega), and 8–12 clones per individual were sequenced using the primer T7 [26]. The results showed that each double-peak represents a polymorphic site, and one to three different sequence types occur in each individual.

### Data analyses

The DNA sequences were aligned with Clustal X [27] and manually adjusted in BioEdit version 7.0.9 [28]. Segregating sites and haplotypes were extracted using DnaSP v5 [29]. All haplotypes have been deposited in GenBank under accession numbers (JQ798299-JQ798389). To estimate the genetic differentiation within species, analysis of molecular variance (AMOVA) was performed based on pairwise differences and haplotype frequencies with Arlequin version 3.1 [30]. Measures of DNA divergence between groups (*F*_ST_) [30] were calculated with Arlequin version 3.1, and the significance was tested using 10 000 permutations. To estimate differentiation between populations, we calculated the values of *G*_ST_ and *N*_ST_ using program HAPLONST [31], with *G*_ST_ taking into account haplotype frequency and *N*_ST_ taking into account both frequency and similarity of the haplotypes. Besides, the *U*-statistic was performed to compare the *G*_ST_ and *N*_ST_, with a higher *N*_ST_ than *G*_ST_ indicating the presence of phylogeographic structure [31]. Phylogenetic relationships among haplotypes were reconstructed by maximum parsimony and Bayesian inference analyses. For the maximum parsimony analysis that was conducted with PAUP version 4.0b10 [32], all characters were treated as unordered and equally weighted, with gaps treated as missing. A heuristic search was performed with 1 000 random addition sequence replicates, tree-bisection-reconnection branch swapping and the MULTREES option. To examine the robustness of clades in the most parsimonious trees, the bootstrap analysis was carried out with 1 000 replicates using the heuristic search options as described above. The Bayesian analysis was conducted with MrBayes version 3.1.2 (nst = 6; rates = invgamma; ngen = 1 000 000) [33], using the best evolutionary model determined by the Akaike Information Criteria implemented in MrModeltest 2.3 [34]. To estimate the significance of difference between the topologies of chloroplast and nuclear gene phylogenies, a Shimodaira–Hasegawa test [35] was performed with PAUP version 4.0b10, using 10,000 bootstrap replicates with the resampling estimated log-likelihood method. The maximum likelihood tree generated from the cpDNA data was compared with the ITS data, and the maximum likelihood tree of the ITS data was assessed relative to the cpDNA data.

To examine the constancy of molecular evolution rate among lineages of the phylogenies, a likelihood ratio test [36] was performed with PAUP version 4.0b10, in which likelihood scores of the trees with and without an enforced molecular clock were compared. Significance was calculated by comparing two times the difference in log likelihoods to a χ^2^ distribution with *n* – 2 degrees of freedom, where *n* is the number of taxa (haplotypes of *M. integrifolia* plus outgroups). When *M. chelidonifolia* served as outgroup, the results of the likelihood ratio tests rejected a clock-like evolution for both cpDNA (P<0.01) and ITS haplotypes (P<0.01). If *M. betonicifolia* and *M. simplicifolia* were chosen as outgroups, the results of the likelihood ratio tests rejected a clock-like evolution for cpDNA (P<0.01) but not for ITS haplotypes (P = 0.096). As no fossil of *Meconopsis* and its close relatives was available, the crown group age (a mean of 9.2 Ma; 95% highest posterior density (HPD): 15.2–3.8 Ma) [37] of *M. integrifolia* and its close relatives was used to date lineage ages of *M. integrifolia* in the cpDNA and nrDNA ITS phylogenies. Given the wide confidence interval of this constraint age, the average nrITS substitution rate for herbaceous plants (1.72–8.34×10^−9^ substitution/site/year, mean = 4.13×10^−9^ subs/site/yr) [38] was also used to estimate the crown group age of *M. integrifolia* and its close relatives. Molecular clock analysis of the age and confidence interval was performed using BEAST v1.6.2 [39], with a Hasegawa-Kishino-Yano plus Gamma site heterogeneity model and a constant population size for the data set. To cover the nrITS substitution rate range (1.72–8.34×10^−9^ subs/site/yr) with a 95% confidence interval for the mean rate, the substitution rate was set to a normally distributed prior with a mean of 4.13×10^−9^ subs/site/yr and a standard deviation of 1.47×10^−9^ subs/site/yr. The Bayesian Markov chain Monte Carlo simulation was run for 20,000,000 generations with a sample frequency of 1000, and the first 2,000,000 generations were discarded as ‘burnin’. Trace file and statistics of estimated parameters were created using Tracer version 1.5 [40]. The final calibrated chronogram and node estimates were edited using FigTree version 1.3.1 [41]. The most recent common ancestor of *M. integrifolia* and its close relatives was dated to 6.20 Ma (95% HPD: 15.23–2.78 Ma) (not shown). Then, a relaxed clock model [42] in BEAST was used to estimate nodal ages and credibility intervals for both cpDNA and nrDNA data sets using *M. chelidonifolia* as outgroup. An uncorrelated lognormal distribution model with a Yule speciation process and a Hasegawa-Kishino-Yano plus Gamma site heterogeneity model was applied in the analyses. To cover the time range (9.2–6.2 Ma) with a 95% confidence interval for the mean age, the crown group age of *M. integrifolia* and its relatives was set to a normally distributed prior with an average of 7.7 Ma and a standard deviation of 0.9 Ma. The remaining settings and procedures were the same as in the nrITS data set described earlier.

## Results

### CpDNA sequence analysis

**Figure 2:**
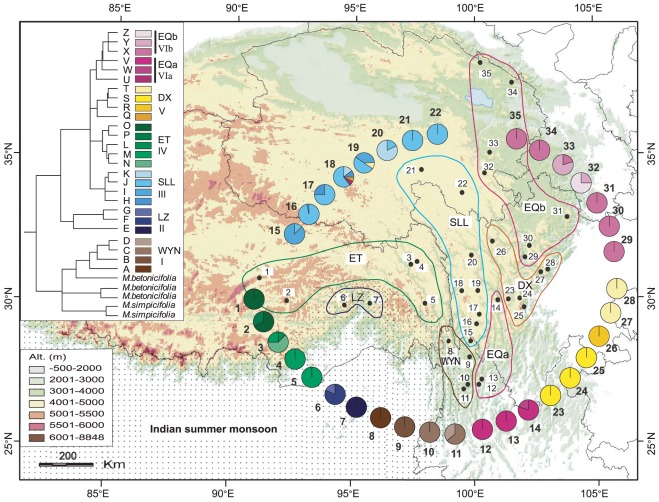
Distribution of the cpDNA haplotypes. Phylogenetic relationships of the haplotypes based on the Bayesian analysis are indicated on the left of the map. Pie charts show the proportions of haplotypes in each population. Doted area indicates the region controlled by the Indian summer monsoon [47].

**Figure 3:**
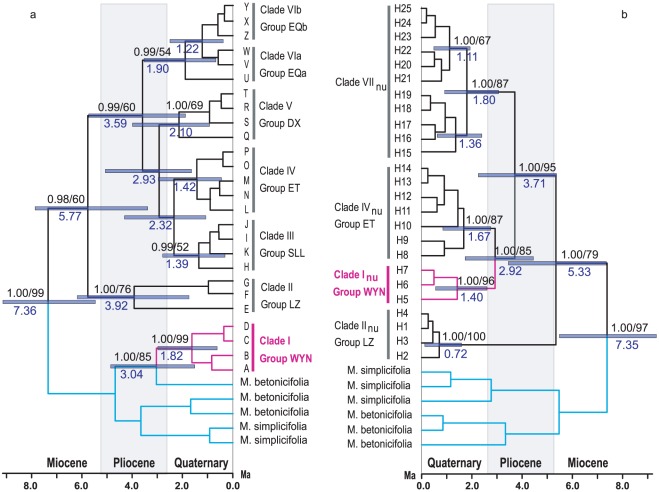
Phylogenetic chronograms of the cpDNA haplotypes (a) and the ITS haplotypes (b) generated from BEAST. Numbers on the branches indicate the Bayesian posterior probabilities (Left) and bootstrap values of 1000 replicates (Right) for the maximum parsimony analysis. Ages of the main clades are shown below the branches and horizontal bars represent estimates for node ages at the 95% highest posterior density.

Forty-five single nucleotide polymorphisms were detected among the 757 individuals of *M. integrifolia*, which defined 26 cpDNA haplotypes (A–Z) (Table S1). For phylogenetic relationships of the haplotypes, the topologies of trees generated by Bayesian (Fig. 2), BEAST (Fig. 3a), and maximum parsimony analyses (not shown) were congruent in the major clades. That is, the 26 haplotypes were clustered into six well supported clades (Fig. 3a), and five of which (Clades II–VI) further formed a strongly supported major clade, whereas clade I is sister to a clade of *M. betonicifolia*.

The 26 cpDNA haplotypes are distributed in seven non-overlapped geographic regions (Fig. 2). The four haplotypes (A, B, C and D) of clade I are exclusively distributed in the population group WYN, and haplotypes E, F and G (clade II) only occur in the two populations (group LZ) located in the north Yarlung Zangbo Grand Canyon. Clade III comprises four haplotypes (H, I, J and K) that only occur in the eight populations (group SLL) along the Shalulishan and Bayankalashan Mountains. Clade IV includes five haplotypes (O, P, L, M and N) that are present exclusively in five populations (group ET) in the central and eastern Tibetan. Haplotypes of clade VI are mainly distributed in the easternmost regions of the Hengduan Mountains (Group EQa & EQb), but the distribution is separated into two parts by the Daxue mountain range and Qionglai mountain range, where the population group DX (Clade V) is located. Although the geographic distribution of haplotypes generally exhibits a lineage-specific pattern (Fig. 2), there are a couple of exceptions. That is, haplotypes Q (clade V) and U (VIa) are only present in population 18 (group SLL, corresponding to clade III), with a low frequency of 7%, respectively (Table S1). Also, haplotype T (clade V) occurs in population 19 (group SLL, corresponding to clade III) with a frequency of 10%, while it is fixed at 100% in populations 27 and 28 of group DX.

Of the studied 35 populations of *M. integrifolia*, twenty-two (62.86%) harbored pure haplotype, ten (28.57%) fixed two haplotypes, and only three (8.57%) had more than two haplotypes. Significant population differentiation was observed, with a *G*_ST_ = 0.843 and a *N*_ST_ = 0.887. The *U*-statistic showed that *G*_ST_ and *N*_ST_ were significantly different from each other (*N*_ST_>*G*_ST_, *U* = 2.73, *P*<0.005). The analysis of molecular variance (AMOVA) revealed that 80.18% of the total variation occurred among groups, 16.82% among populations, and 3.00% within populations (Table 1). Pairwise *F*_ST_ among geographic regions varied from 0.7397 to 0.9054, suggesting a high genetic differentiation between the geographic regions (Table 2).

**Table 1:** Hierarchical analysis of molecular variance (AMOVA) for populations of *Meconopsis integrifolia* based on chloroplast DNA haplotypes.

Source of variation	d. f.	Sum of squares	Variance components	Percentage of variation (%)
Among groups	6	1798.661	2.742	80.18
Among populations	28	350.282	0.575	16.82
Within populations	722	74.098	0.102	3.00
Total	756	2223.041	3.419	

**Table 2:** Pairwise comparisons of *F*_ST_ (chloroplast DNA haplotypes) between seven geographic regions of *Meconopsis integrifolia*.

	ET	LZ	WYN	EQa	EQb	SLL	DX
ET							
LZ	0.7397*						
WYN	0.8463*	0.7485*					
EQa	0.8632*	0.7554*	0.8655*				
EQb	0.8787*	0.8455*	0.9054*	0.7255*			
SLL	0.7487*	0.7809*	0.8799*	0.8390*	0.8673*		
DX	0.8095*	0.7932*	0.8788*	0.8889*	0.8984*	0.8116*	

* *P*<0.001.

### NrDNA ITS sequence analysis

Twenty-five nrDNA ITS haplotypes (alleles) were detected from 151 individuals representing the seven population groups (Table S1). Phylogenetic relationships of the haplotypes are consistent between the Bayesian tree (not shown), maximum parsimony tree (not shown), and the tree generated by the BEAST analysis (Fig. 3b). Monophyly of the 25 haplotypes is supported by the high bootstrap value and posterior probability (Fig. 3b), and four major clades (I_nu_, II_nu_, IV_nu_ and VII_nu_) with high bootstrap and posterior probability values were recognized. In the ITS tree (Fig. 3b), the group WYN (Clade I_nu_) is a natural group of *M. integrifolia* rather than being nested into *M. betonicifolia* as in the cpDNA tree (Fig. 3a). A significant topological incongruence between the two trees was supported by the Shimodaira-Hasegawa test (P<0.001). Similar to the distribution of the cpDNA haplotypes (Fig. 3a), the clade I_nu_ and II_nu_ of the ITS haplotypes are exclusively restricted to population groups WNY and LZ (corresponding to clades I and II in the cpDNA phylogeny), respectively (Table S1). Clade IV_nu_ includes seven haplotypes (H8–H14) that only occur in population group ET (corresponding to clade IV in the cpDNA phylogeny), but two haplotypes (H18, H19) of clade VII_nu_ occur in population 2 of this group. The monophyletic relationships of groups SLL, DX and EQ were not supported by the nrDNA ITS analysis.

### Molecular dating

When the crown group age of *M. integrifolia* and its close relatives was set to 7.7(±1.5) Ma as a constraint, the most recent common ancestor of *M. integrifolia* was dated to about 5.77 Ma (95% highest posterior density (HPD): 7.86–3.38 Ma) based on the cpDNA haplotypes (Fig. 3a) and around 5.33 Ma (95% HPD: 7.35–3.45 Ma) based on the ITS haplotypes (Fig. 3b). The crown age of the oldest clade II in the cpDNA phylogeny was dated to 3.92 Ma (95% HPD: 6.19–1.74 Ma), while the divergence times of the other main clades (I, III–VI) were estimated to range from 3.59 to 2.32 Ma. The divergence between clade I_nu_ and clade IV_nu_ was dated to 2.92 Ma (95% HPD: 4.45–1.73 Ma), and that between clade VII_nu_ and its sister clade was estimated to be 3.71 Ma (95% HPD: 5.37–2.26 Ma).

## Discussion

### Marked phylogeographic structure and genetic differentiation among highly fragmented populations of *M. integrifolia* and its implications for plant speciation in the QTP

It is well known that complex topography could have great effects on the patterns of population genetic variation in space and time [10]. To date, more than ten plant species in the QTP have been phylogeographically studied [12]. The common scenario is that the genetic variation was structured as a result of the interglacial re-colonization of the QTP platform from multiple glacial refugia, as retrieved by the mtDNA and/or cpDNA analyses of different kinds of plants. This conclusion was further supported by the perfect congruence of the mtDNA, cpDNA and nrDNA analyses on the conifer *Tsuga dumosa* [14]. However, few studies revealed a pattern of population differentiation associated with the subtle landscape feature of the QTP [12], given that the majority of surveyed species are either conifers with strong dispersal ability or dominant species with a wide distribution on the plateau.

The distribution pattern of the 26 cpDNA haplotypes detected in *M. integrifolia* indicates a subtle geographic genetic structure and remarkable genetic differentiation among lineage-specific population groups of the species (Fig. 2), which is also supported by the results of analysis of molecular variance (AMOVA) (Table 1), pairwise *F*_ST_ (Table 2), and the *U*-statistic of *G*_ST_ and *N*_ST_. As shown in Fig. 2, seven lineage-specific groups are well isolated in the eastern QTP. Three of them (ET, SLL & DX) are distributed exclusively in three mountain ranges with average altitudes above 4000 m above sea level, and the other four groups (WYN, LZ, EQa & EQb) inhabit in the edge of the QTP at relatively lower altitudes. That is, group ET includes five populations along the Nyainqentanglha Mountains, group SLL along the Shalulishan & Bayankalashan Mountains, and group DX in the Daxue Mountains & Qionglai Mountains. The exceptions, i. e., haplotypes U and Q in population 18 (group SLL) and haplotype T in population 19 (group SLL), might be resulted from gene flow from neighboring groups DX and EQa by seed dispersal in a low frequency. Analysis of the ITS data set also indicates a lineage-specific pattern of haplotype distribution, such as the three clades I_nu_, II_nu_, and VI_nu_ that correspond to groups WYN, LZ and ET, respectively (Fig. 3b). However, three lineage-specific groups (SLL, DX, and EQ) revealed in the cpDNA analysis are not supported by the ITS analysis. This could be attributed to the difference in gene flows through seed and pollen [43], and may suggest that the mountain ranges could have acted as dispersal corridors for the native groups, although these corridors were isolated by unfavorable habitats, especially the heterogeneous environment along the deep-incised valleys (Fig. 1, 2). On the other hand, these three mountains could also have blocked the spread of the four peripheral population groups (WYN, LZ, EQa & EQb) at relatively lower altitudes. The subtle phylogeographic structure among lineage-specific groups clearly indicates how the complex landscape, particularly the mountain ranges, has greatly affected the evolutionary history of the QTP plants (Figs. 1, 2).

Moreover, individuals in relatively low altitudes (3000–4000 m) often inhabit in forest gaps or under shrub, a habitat quite different from the alpine scree at higher altitudes of the QTP. The great population genetic differentiation (*F*_ST_) (Table 2) and extensive morphological variation [19] in *M. integrifolia* suggest that the population groups have been isolated for quite a long time, and the low genetic variation within population (3.00% from the AMOVA analysis) indicates that populations of the species have experienced genetic drift. An increasing number of studies indicated that habitat fragmentation could play important roles in the divergence and speciation for plants [1], [2], [3]. Allopatric speciation is prone to occur in fragmented populations [1], whereas genetic drift resulting from the reduction in population size during habitat fragmentation could erase genetic diversity in isolated populations, leading to the decrease of population viability and extinction of species [1], [3]. Our molecular clock estimate based on cpDNA suggests that the divergence of main clades of *M. integrifolia* occurred during late Miocene (5.77 Ma) to early Quaternary (2.32 Ma) (Fig. 3a), a time span that is generally congruent with that estimated based on the ITS haplotypes (5.33–2.92 Ma) (Fig. 3b). The estimated divergence times of the main lineages are much earlier than the oldest Quaternary glaciation (about 0.71 Ma) [44] in the QTP. Therefore, the Quaternary climatic oscillations could not have contributed greatly to the divergence of the main clades of *M. integrifolia*, although it can not be completely ruled out. It is believed that the QTP reached an elevation like present at about 8.0 Ma [45], but was lowered by the following extensive faulting, and its recent rapid uplift occurred at around 3.6 Ma [46]. So, the divergence of the main clades of *M. integrifolia* during early to middle Quaternary is strongly correlated to the recent rapid uplift of the plateau [46]. That is, the rapid uplift of the QTP led to the strong fragmentation of the original continuous distribution of *M. integrifolia*, and the heterogeneous habitats along an elevation gradient hindered the dispersal of the species among the “sky islands”, considering its limited dispersal ability (with insect-pollinated flower and gravity-dispersed seed). Given the uncertainty with the constraint age used in the present study, this scenario should be accepted with caution. However, our inference is consistent with previous reports that lineage divergence and speciation of plants in the QTP could be attributed to the topography changes resulting from the rapid uplift of the plateau [16], [17], [18]. As discussed above, the strong phylogeographic structure and great genetic differentiation among population groups of *M. integrifolia* may imply that habitat fragmentation arising from the uplift of the QTP has played a key role in speciation and extinction of the QTP plants.

Another interesting finding is the rough correlation between the haplogroups' distribution and the regional climate in the QTP (Fig. 2). A typical pattern revealed from many previous studies [12] is that primitive lineages or ancestral haplotypes were distributed in the east edge of the QTP (refugia), from where northwestward re-colonization occurred. This pattern could be mostly attributed to the Quaternary climatic oscillations in the QTP [12]. However, evidence from the cpDNA and nuclear DNA (ITS) data set indicate that the older group (LZ) of *M. integrifolia* only occurs in southeast Tibet, with the distributions of younger groups (WYN, ET, SLL, DX, and EQ) extending northeastward in the eastern QTP (Fig. 2, 3). There is palaeo-climatic evidence that the climatic shifts of the QTP and adjacent regions have been controlled by the Indian summer monsoon (southwest Asia monsoon) arising from the rapid uplift of the QTP [47], [48]. The Indian summer monsoon brings heavy summer rainfall for the south edge of the Himalayas, but leads to sharp decrease of precipitation in other area of the QTP due to the high topographic barrier (Fig. 2) [47]. It is believed that the climatic divergence between the south edge and the other region of the QTP has developed since late Miocene when the Asia monsoon system initiated [48]. The initial divergence of *M. integrifolia* dated to around 5.77 (95% HPD: 7.86–3.38) Ma based on cpDNA or 5.33 (95% HPD: 7.35–3.45) Ma based on ITS seems to have occurred synchronously with the climatic divergence in the QTP [48]. The phylogeographic structure associated with the regional climate suggests that climatic divergence driven by the rapid uplift of the QTP likely led to the initial divergence of *M. integrifolia*.

### Incongruence between cpDNA and nrDNA ITS haplotype phylogenies

Incongruence between phylogenies of nuclear and plastid DNA is generally caused by convergent evolution, lineage sorting, or hybridization/introgression [49]. Conflict between gene trees is a common phenomenon in plants, but few studies of the QTP plants revealed it and the underlying mechanism at the population level [18]. In the present study, we found that the phylogenetic position of group WYN of *M. integrifolia* is very different between the cpDNA (clade I) and ITS (clade I_nu_) phylogenies constructed based on population sampling (Fig. 3), and the significance of this topological incongruence is also supported by the Shimodaira–Hasegawa test. Given the non-coding regions we used, convergent evolution should not be responsible for the reticulate evolution in *M. integrifolia*. Although incomplete lineage sorting could result in the stochastic fixation of ancestral polymorphisms in descendant populations, it is not supported by the strong geographic partition and early divergence of the cpDNA haplotypes, and the relatively antiquated ITS haplotypes of the population group WYN. However, hybridization frequently occurs in plants [49], and putative natural hybrids were reported in the genus *Meconopsis* [50]. Also, the alternation of topography resulting from the uplift of the QTP could have led to the contact of species originally distributed far from each other, providing opportunities for interspecific hybridization. So it is quite likely that the reticulate evolution was caused by hybridization between *M. integrifolia* and *M. betonicifolia*, and the chloroplast DNA of the latter was captured by the population group WYN of the former.

Although natural hybrids between *M. integrifolia* and its close relatives have never been reported so far, the cultivated hybrids ×*M. sarsonsii* (*M. integrifolia*×*M. betonicifolia*) and ×*M. harleyana* (*M. integrifolia*×*M. simplicifolia*) were reported about eighty years ago [50]. The hybrids could occur spontaneously or by deliberated crossing in the Royal Botanic Garden, Edinburgh. It is interesting that the flowers of the two hybrids were yellow as that of its parent *M. integrifolia* [50]. As a result, ×*M. harleyana* was identified as a yellow-flowered variety of *M. simplicifolia* rather than a hybrid, since it was often difficult to distinguish hybrids of *Meconopsis* only based on morphological characters [50]. In fact, the taxonomic status of the putative hybrid (group WYN) has confused botanists for about one hundred years owning to the intermediate morphological characters [19], [50], [51], [52], and it was even treated as a new species *M. pseudointegrifolia*, a variety of *M. integrifolia* or *M. pseudointegrifolia* [50], [52]. According to the present study, the most possible parents of the hybrid are *M. integrifolia* and *M. betonicifolia* (Fig. 1, 3), but further investigation is necessary to corroborate this hypothesis considering the insufficient investigation of the genus in the geographically challenging region (QTP).

Field investigation found that the distributions of *M. integrifolia* and *M. betonicifolia* seldom overlap, and the former often inhabits at higher altitudes than the latter on the same mountain. Although the clade I includes four haplotypes widely distributed in northwest Yunnan (Fig. 2), the overlap distribution of the two species was only found in the Laojunshan Mountain. So it is unlikely that the hybridization has occurred recently, and the molecular dating also supports an ancient hybridization between the two species (Fig. 3).

Hybridization plays a great role in plant speciation, especially for groups with a high number of ployploids [53]. The environmental and habitat heterogeneity are though to promote the rapid speciation by hybridization. Our phylogeographic analysis of *M. integrifolia* suggests that geographic isolation and hybridization could be two important mechanisms responsible for the divergence and speciation of *Meconopsis*, a species-rich genus with complex polyploids [20], and even for speciation in other plant groups of the QTP.

## Supporting Information

Table S1 **Geographic origins, sample sizes, haplotypes and their frequencies of the 35** ***Meconopsis integrifolia*** **populations studied.**
(DOC)
