# Supplementary material for: Great Genetic Differentiation among Populations of Meconopsis integrifolia and Its Implication for Plant Speciation in the Qinghai-Tibetan Plateau
Source: PLoS One. 2012 May 10;7(5):e37196. doi: 10.1371/journal.pone.0037196 (PMC3349641; doi:10.1371/journal.pone.0037196)
Supplement: Table S1 — Geographic origins, sample sizes, haplotypes and their frequencies of the 35 Meconopsis integrifolia populations studied. (DOC) [file pone.0037196.s001.doc]

**Table S1.** Geographic origins, sample sizes, haplotypes and their frequencies of the 35 *Meconopsis integrifolia* populations studied.

| Group | Population | | Code | Longitude | Latitude | Altitude | Number  cpDNA ITS | | Haplotypes (Frequencies, %)  cpDNA ITS | |
| --- | --- | --- | --- | --- | --- | --- | --- | --- | --- | --- |
| ET | 1 | Mt. Nyainqentanglha, T | NMC | 30.679 | 91.103 | 5200 | 23 | 8 | O(100) | H10(75),H8(12.5),H9(12.5) |
|  | 2 | Mt. Milashan, T | MLS | 29.850 | 92.340 | 4885 | 22 | 5 | O(68.2),P(31.8) | H19(80),H18(20) |
|  | 3 | Changdu, T | CD | 31.153 | 97.428 | 4300 | 16 |  | L(12.5),M(25),N(62.5) |  |
|  | 4 | Changdu,T | CDSD | 31.281 | 97.332 | 4587 | 24 | 8 | M(100) | H12(100) |
|  | 5 | Mt. Dongdashan,T | DDS | 29.712 | 98.000 | 5088 | 25 | 8 | M(100) | H14(75)H11(12.5),H13(12.5) |
| LZ | 6 | Mt. Sejilashan,T | SJLS | 29.613 | 94.654 | 4510 | 22 | 8 | F(81.8),G(18.2) | H1(73),H2(9),H3(9),H4(9) |
|  | 7 | Mt. Galongla, T | BMMT | 29.768 | 95.689 | 4000 | 17 |  | E(100) |  |
| WYN | 8 | Mt. Baimangxueshan, YN | BMXS | 28.411 | 98.991 | 3887 | 20 | 8 | A(100) | H5(100) |
|  | 9 | Xianggelila, YN | XZD | 27.543 | 99.920 | 3540 | 15 | 8 | B(100) | H6(100) |
|  | 10 | Mt. Laojunshan, YN | LJS | 26.635 | 99.717 | 3875 | 23 | 8 | C(100) | H7(100) |
|  | 11 | Mt. Laojunshan, YN | LJSB | 26.588 | 99.667 | 3850 | 22 |  | C(64),D(36) |  |
| EQa | 12 | Mt.Yulongxueshan, YN | YL | 27.054 | 100.195 | 3834 | 21 | 8 | V(100) | H15(100) |
|  | 13 | Mt.Yulongxueshan, YN | YLB | 27.120 | 100.261 | 3850 | 21 |  | V(100) |  |
|  | 14 | Mt. Jianziwanshan. SC | JZWS | 30.007 | 100.862 | 4345 | 20 | 8 | V(80),W(20) | H16(100) |
| SLL | 15 | Xianggelila, YN | ZDGZ | 28.133 | 99.891 | 4168 | 23 | 8 | J(13),I(87) | H15(100) |
|  | 16 | Mt. Wumingshan, SC | WMS | 29.146 | 100.087 | 4595 | 29 | 8 | J(97),I(3) | H22(100) |
|  | 17 | Mt. Hanzishan, SC | BHZ | 30.281 | 99.555 | 4658 | 12 |  | J(75),H(25) |  |
|  | 18 | Mt. Tuershan, SC | DHZ | 29.549 | 100.193 | 4586 | 28 |  | J(61),K(14),H(11),Q(7),U(7) |  |
|  | 19 | Mt. Langdashan, SC | LDS | 30.233 | 100.264 | 4457 | 10 |  | J(50),I(40),T(10) |  |
|  | 20 | Mt. Zhuodalashan, SC | GZ | 31.404 | 99.965 | 4804 | 22 |  | J(18),K(82) |  |
|  | 21 | Mt. Bayankalashan, QH | BY | 34.398 | 97.995 | 4443 | 25 | 8 | J(100) | H23(100) |
|  | 22 | Dari, QH | DR | 33.747 | 99.640 | 4026 | 21 | 7 | J(100) | H23(100) |

Abbreviations: SC, Sichuan Province; YN, Yunnan Province; QH, Qinghai Province; T, Tibet Autonomous Region.

**Table S1.** (continued)

| Group | Population | | Code | Longitude | Latitude | Altitude | Number | | Haplotypes (Frequencies, %) | |
| --- | --- | --- | --- | --- | --- | --- | --- | --- | --- | --- |
|  |  |  |  |  |  |  | cpDNA ITS | | cpDNA ITS | |
| DX | 23 | Mt. Gaoersishan, SC | YJ | 30.066 | 101.327 | 4224 | 23 |  | S(100) |  |
|  | 24 | Kangding, SC | KDYL | 29.914 | 101.993 | 3805 | 19 |  | S(100) |  |
|  | 25 | Mt. Zheduoshan, SC | ZD | 30.050 | 101.750 | 4210 | 25 | 6 | S(100) | H19(100) |
|  | 26 | Seda, Sichuan | SD | 31.751 | 100.757 | 4246 | 19 |  | R(100) |  |
|  | 27 | Mt. Balangshan, SC | BLS | 30.911 | 102.895 | 4480 | 20 | 8 | T(100) | H22(12.5),H17(87.5) |
|  | 28 | Mt. Jiajinshan, SC | JJS | 30.871 | 102.683 | 4069 | 23 |  | T(100) |  |
| EQb | 29 | Mt. Mengbishan, SC | MBS | 31.706 | 102.298 | 4018 | 26 | 7 | X(100) | H25(85.7),H24(14.3) |
|  | 30 | Mt. Xuebaoding, SC | SP | 32.750 | 103.690 | 4005 | 24 | 8 | X(100) | H21(100) |
|  | 31 | Mt. Yakoushan, SC | HY | 32.650 | 102.367 | 4080 | 24 |  | X(100) |  |
|  | 32 | Maqin, QH | DW | 34.244 | 100.243 | 4011 | 27 | 6 | Y(22),Z(88) | H23(33.3),H20(66.7) |
|  | 33 | Tongde, QH | TD | 34.999 | 100.843 | 4105 | 28 | 8 | X(25),Y(75) | H20(100) |
|  | 34 | Menyuan, QH | MY | 37.670 | 101.438 | 3649 | 22 |  | X(100) |  |
|  | 35 | Qilian, QH | QL | 38.129 | 100.234 | 3500 | 16 |  | X(100) |  |
